# Left Flank Pain

**DOI:** 10.5811/westjem.2015.1.25076

**Published:** 2015-02-26

**Authors:** Thomas M. Nappe, Shawn M. Quinn

**Affiliations:** Lehigh Valley Hospital, Department of Emergency Medicine, Allentown, Pennsylvania

An 18-year-old female presented to the emergency department with three days of worsening left flank pain. Past medical history included asymptomatic bacteriuria. She denied prior similar episodes or inciting events, and was currently being treated with trimethoprim sulfamethoxazole by an urgent care center for a urinary tract infection, although she denied having any urinary symptoms. Upon evaluation, she was found to be in severe pain, refractory to multiple doses of opioids. Her examination revealed fever, mild tachycardia and tachypnea, and clear lungs, with significant tenderness to palpation on her left flank. Initial laboratory evaluation showed a leukocytosis and bacteriuria.

A computed tomography of the abdomen and pelvis was ordered with intravenous contrast to rule out pyelonephritis and infected renal calculus. It revealed Figure 1, which shows an inflamed pulmonary sequestration in the posteromedial left lung base, with a surrounding pleural effusion. An independent arterial blood supply branching off of the descending thoracic aorta was later confirmed with angiography, shown in Figure 2, which further revealed infarction secondary to torsion.

A pulmonary sequestration is a congenital malformation of independent, nonfunctioning, pulmonary tissue that does not communicate with the tracheobronchial tree and often has its own independent systemic blood supply.^1^–^4^ Both the location and associated effusion in this image indicate an extralobular pulmonary sequestration, which is defined by the presence of its own pleura.^1^,^2^,^5^ Presentation can occur at various ages and can include recurrent pneumonia, hemoptysis and pain, and persistent or severe symptoms may indicate surgical resection.^5^,^6^

## Figures and Tables

**Figure 1:**
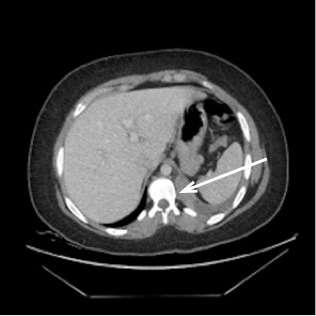
Computed tomography image of thorax with pulmonary sequestration present at left base.

**Figure 2:**
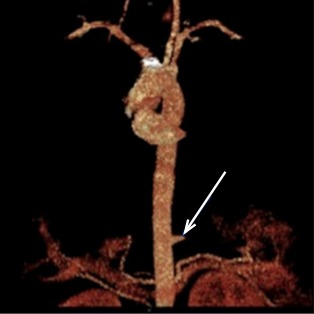
Angiography showing arterial blood supply to pulmonary sequestration with abrupt lack of flow due to torsion.
